# Mallory-Denk bodies and hepatocellular senescence: a causal relationship?

**DOI:** 10.1007/s00428-024-03748-1

**Published:** 2024-01-30

**Authors:** Helmut Denk, Peter M. Abuja, Kurt Zatloukal

**Affiliations:** Diagnostic and Research Institute of Pathology, Diagnostic & Research Center of Molecular Biomedicine, Medical University of Graz, Neue Stiftingtalstrasse 6, A-8010 Graz, Austria

**Keywords:** Steatohepatitis, Chronic cholangiopathy, Keratins, Mallory-Denk bodies, Cellular senescence

## Abstract

Mallory-Denk bodies (MDBs) are hepatocellular cytoplasmic inclusions, which occur in certain chronic liver diseases, such as alcohol-related (ASH) and metabolic dysfunction-associated (MASH) steatohepatitis, copper toxicosis, some drug-induced liver disorders, chronic cholangiopathies, and liver tumors. Our study focused on the expression of the senescence markers p21^WAF1/cip1^ and p16^INK4a^ in hepatocytes containing MDBs in steatohepatitis, chronic cholangiopathies with fibrosis or cirrhosis, Wilson’s disease, and hepatocellular carcinomas. Cytoplasm and nuclei of MDB-containing hepatocytes as well as MDB inclusions, except those associated with carcinoma cells, were strongly p16-positive, p21-positive, as well as p21-negative nuclei in MDB-containing hepatocytes which were observed whereas MDBs were p21-negative. Expression of the senescence marker p16 suggests that MDB formation reflects an adaptive response to chronic stress resembling senescence with its consequences, i.e., expression of inflammation- and fibrosis-prone secretome. Thus, senescence can be regarded as “double-edged sword” since, on the one hand, it may be an attempt of cellular defense, but, on the other, also causes further and sustained damage by inducing inflammation and fibrosis related to the senescence-associated secretory phenotype and thus progression of chronic liver disease.

## Introduction

Mallory-Denk bodies (MDBs) are hepatocellular cytoplasmic inclusions with filamentous ultrastructure, which occur in certain chronic liver diseases, such as alcohol-related (ASH) and metabolic dysfunction-associated (MASH) steatohepatitis, copper toxicosis, some drug-induced liver diseases, chronic cholangiopathies with fibrosis/cirrhosis, and liver tumors [1–3]. They can be experimentally induced in mouse liver by prolonged griseofulvin or 3,5-diethoxycarbonyl-1,4-dihydrocollidine (DDC) feeding, but may also arise in aged ferrochelatase-deficient (fch/fch) porphyric mice, keratin(K)18−/− mice (in contrast to K8−/− mice), and K8-overexpressing mice [3, 4]. Thus, chronic intoxication and cellular stress in association with advanced age are common principles indicating that MDB formation may result from long-lasting metabolic disturbances [5]. In these pathologic situations MDBs are not universally present in human or mouse hepatocytes but prefer cells in centrilobular areas or at the periphery of cirrhotic nodules [1, 2]. The fact that MDBs are usually detected in only a minority of hepatocytes, particularly in human diseases, suggests that a special constellation of metabolic alterations in the individual cell accounts for their development. The major constituents of MDBs are phosphorylated and misfolded Ks (especially K8), ubiquitin, sequestosome 1/p62 (p62), and heat shock proteins; Ks are cross-linked by transglutaminase action (for which K8 is a preferred substrate), as revealed by immunohistochemistry and chemical analyses [1–3]. MDB-like protein aggregates can also be produced in vitro by transfection of these components into tissue culture cells [6, 7]. Thus, we are dealing with misfolded proteins, which are produced in response to chronic stress and apparently inadequately degraded followed by accumulation and aggregation. The morphologic appearance of MDB-containing hepatocytes suggests that they are viable; they are enlarged (ballooned) with disarranged or even deficient K intermediate filament cytoskeleton and increased nuclear and nucleolar size suggesting increased protein synthesis [2, 8]. Indeed, in situ hybridization revealed elevated K synthesis in MDB-containing hepatocytes as consequence of chronic DDC intoxication of mice [9]. Chronic stress, particularly of oxidative nature, has been shown to be a feature of MDB-expressing disorders in humans and animals and is also known to contribute to cellular senescence [4, 10–13]. It was, therefore, the aim of our study to explore in human livers at the tissue level whether features of senescence, indicated by the expression of p16^INK4a^ (p16) and p21^WAF1/cip1^ (p21), are associated with the pathogenesis of MDBs and related pathologic findings.

## Material and methods

Liver specimens, obtained by surgery or biopsy, in which MDB-containing hepatocytes were present, were selected from the diagnostic work of the Diagnostic and Research Institute of Pathology, Medical University of Graz, Austria, and provided by the Biobank Graz. They included ASH (10 cases), MASH (11 cases), chronic cholangiopathies (6 cases), Wilson’s disease (WD; 3 cases), and hepatocellular carcinomas (HCC; 4 cases) (Table 1). ASH, MASH, and WD were staged according to Kleiner et al. [14] and chronic cholangiopathies according to Ludwig and Batts [15]. HCCs resembled the steatohepatitic variant [16]. The study was approved by the Ethics Committee of the Medical University of Graz, Austria. The material was fixed in 10% neutral buffered formaldehyde solution, embedded in paraffin by routine procedures, and 5-μm-thick sections were stained after removal of paraffin with hematoxylin–eosin (H&E) and chromotrope aniline blue (CAB) for light microscopy [17]. For immunohistochemistry, antibodies to p16 (CINtec Ventana, Tucson, USA, E6H4, mouse monoclonal, IgG 2a; ready to use; incubation time 20 min) and to p21 (Dako, Glostrup, DK, Clone SX118; monoclonal mouse antihuman, IgG1, kappa; dilution 1:20; incubation time 30 min) were used as reported previously [17]. Some specimens were in addition immunostained using antibodies to ubiquitin (DakoCytomation, Glostrup, DK, Z 0458, rabbit polyclonal; dilution 1:500, incubation time 20 min) for better identification of MDBs. Antigen retrieval was achieved by pretreatment of the sections with CC1 mild (Cell Signaling Technology; for p16), microwave Dako 9.0 (40 min; for p21), and Dako Omnis low pH (for ubiquitin). Detection kits and chromogens were Omnis ENV Flex DAB (for p16), Dako ENV DAB (for p21), and Dako FlexEnvision Kit (for ubiquitin). As negative controls, the specific antibodies in the first layer were replaced by the buffer used for dilution of the specific primary antibody. p16 expression in MDBs was assessed as positive or negative. p21 expression was semiquantitatively graded as follows: grade 1 = 1–10, grade 2 = 11–20, grade 3 ≥ 20 p21-positive hepatocyte nuclei/per high power field (×400; in each case 10 high power fields were evaluated). Immunostainers used in the assays were from Dako or Ventana.

**Table 1:** Patients, histologic diagnoses, p16 expression in Mallory-Denk bodies (MDBs), and p21 expression in hepatocyte nuclei

Patients	Age (years)	Gender	Histologic diagnosis/fibrosis	p16 expression in MDBs	p21 expression (grade)
1	61	M	ASH/F2	Positive	2
2	40	F	ASH/F4	Positive	1
3	68	M	ASH/F3	Positive	3
4	67	F	ASH/F3	Positive	2
5	67	F	ASH/F3	Positive	3
6	49	F	ASH/F4	Positive	3
7	47	F	ASH/F3	Positive	3
8	53	M	ASH/F4	Positive	3
9	63	M	ASH/F4	Positive	4
10	45	M	ASH/F4	Positive	3
11	69	F	MASH/F3	Positive	2
12	53	M	MASH/F3	Positive	3
13	53	F	MASH/F2	Positive	3
14	64	M	MASH/F3	Positive	3
15	74	F	MASH/F2	Positive	3
16	47	M	MASH/F3	Positive	3
17	72	F	MASH/F3	Positive	3
18	65	M	MASH/F3	Positive	3
19	53	M	MASH/F3	Positive	3
20	82	F	MASH/F4	Positive	3
21	56	F	MASH/F3	Positive	3
22	47	M	PSC/4	Positive	3
23	69	F	PBC/3	Positive	3
24	62	M	PBC/4	Positive	2
25	62	F	PBC/4	Positive	1
26	74	F	SBC	Positive	3
27	56	M	SBC	Positive	1
28	54	M	WD/F3	Positive	1
29	54	M	WD/F3	Positive	2
30	63	M	WD/F4	Positive	1
31	72	M	HCC/ASH/F4	Negative/positive	1/2
32	65	M	HCC/MASH/F4	Negative/positive	1/2
33	76	M	HCC	Negative	3
34	71	M	HCC	Negative	3

Gender: *M*, male; *F*, female

*MDBs*, Mallory-Denk bodies

*ASH/F*, alcohol-related steatohepatitis/fibrosis stage according to Kleiner et al. [14]

*MASH/F*, metabolic dysfunction-associated steatohepatitis/fibrosis stage (according to Kleiner et al. [14])

*PBC/stage*, primary biliary cholangitis/stage (according to Ludwig and Batts [15])

*PSC/stage*, primary sclerosing cholangitis/stage (according to Ludwig and Batts [15])

*SBC*, secondary biliary cirrhosis due to prolonged mechanical bile duct obstruction

*WD/F*, Wilson’s disease/fibrosis stage according to Kleiner et al. [14]

HCC (hepatocellular carcinoma)/surrounding non-neoplastic liver

Grading of p21-positive hepatocyte nuclei: number of positive nuclei per high power field (×400); in each case evaluation of 10 high power fields: 1 = between 1 and 10; 2 = between 11 and 20; 3 ≥ 20

## Results

### Steatohepatitis (Fig. 1)

Light microscopically, the specimens resembled the well-known features of ASH and MASH with variable grades of macrovesicular steatosis, fibrosis, or cirrhosis [1, 2]. MDBs were usually abundant, well-formed, homogeneous, and strongly stained in ASH, whereas in MASH, they were often less numerous, less distinct, and more granular. MDB-containing hepatocytes were predominantly located in the periseptal area in the fibrotic and cirrhotic liver. They were rounded (ballooned) with clearing of the cytoplasm, enlarged nuclei, and prominent nucleoli and often surrounded or sometimes even penetrated by neutrophils and mononuclear cells (satellitosis).

**Fig. 1:**
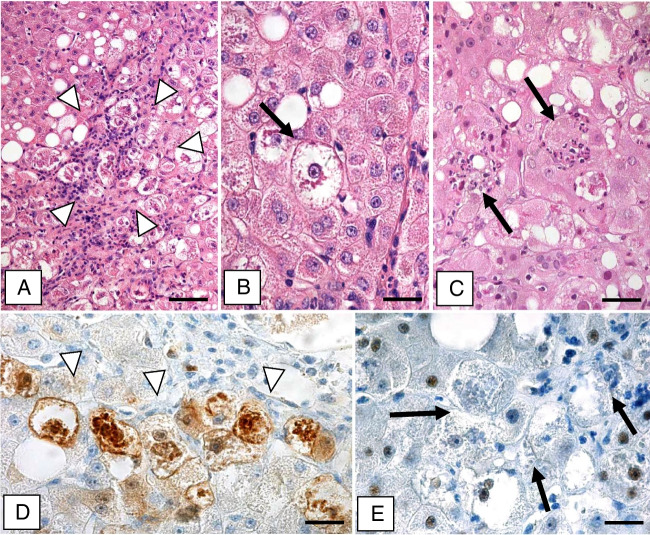
Alcohol-related liver cirrhosis with MDB formation. **a** Area with ballooned hepatocytes containing MDBs (arrow-heads). **b** Ballooned hepatocyte containing MDB shown at higher magnification (arrow). **c** MDB-containing hepatocytes surrounded by neutrophil granulocytes (satellitosis; arrows). **d** p16-positive hepatocytes and MDBs (immunohistochemistry using antibodies to p16; arrow-heads). **e** MDB-containing hepatocytes lack p21 (immunohistochemistry using antibodies to p21; arrows). Bars: **a** 80 μm; **b**, **d**, **e** 20 μm; **c** 15 μm

Antibodies to p16 revealed faint positive immunostaining of the cytoplasm and nuclei of a variable number of hepatocytes with predominance in acinar zone 1 or at the periphery of parenchymal nodules. Hepatocytes in some small cirrhotic nodules were even totally p16-positive. In MDB-containing hepatocytes, however, the intensity of p16 immunostaining was strong involving cytoplasm, nuclei, as well as MDBs. In addition, predominantly, ductules with branching (“atypical”) morphology revealed nuclear and cytoplasmic p16 immunostaining, although p16-negative ductules were also observed. The p16-positive ductules were often in proximity to hepatocytes expressing p16 in their cytoplasm and nuclei. Epithelial cells of regular interlobular bile ducts were either p16-negative or only few displayed nuclear and cytoplasmic positivity. p16-positive elongated sinusoidal cells were also present.

Antibodies to p21 revealed in most cases pronounced nuclear immunostaining in hepatocytes (see grading in Table 1). Some, but not all, MDB-containing hepatocytes displayed p21-positive nuclei, whereas MDBs consistently remained negative. In addition, a variable number of ductules displayed p21-positive nuclei, whereas interlobular bile ducts of different sizes with regular lumina were negative.

### Chronic cholangiopathies in fibrotic or cirrhotic stage (Fig. 2; Table 1)

The specimens studied included primary biliary cholangitis, primary sclerosing cholangitis, and secondary biliary cirrhosis (due to prolonged mechanical bile duct obstruction). Morphology and histological stage of the specimens corresponded to that published by Ludwig and Batts [15] with variable degrees of ductular reaction and lymphocytic infiltration, moderate ductopenia, occasional periductal fibrosis, and obliteration of larger bile ducts. Some periseptal hepatocytes were enlarged with weakly stained cytoplasm containing MDBs.

**Fig. 2:**
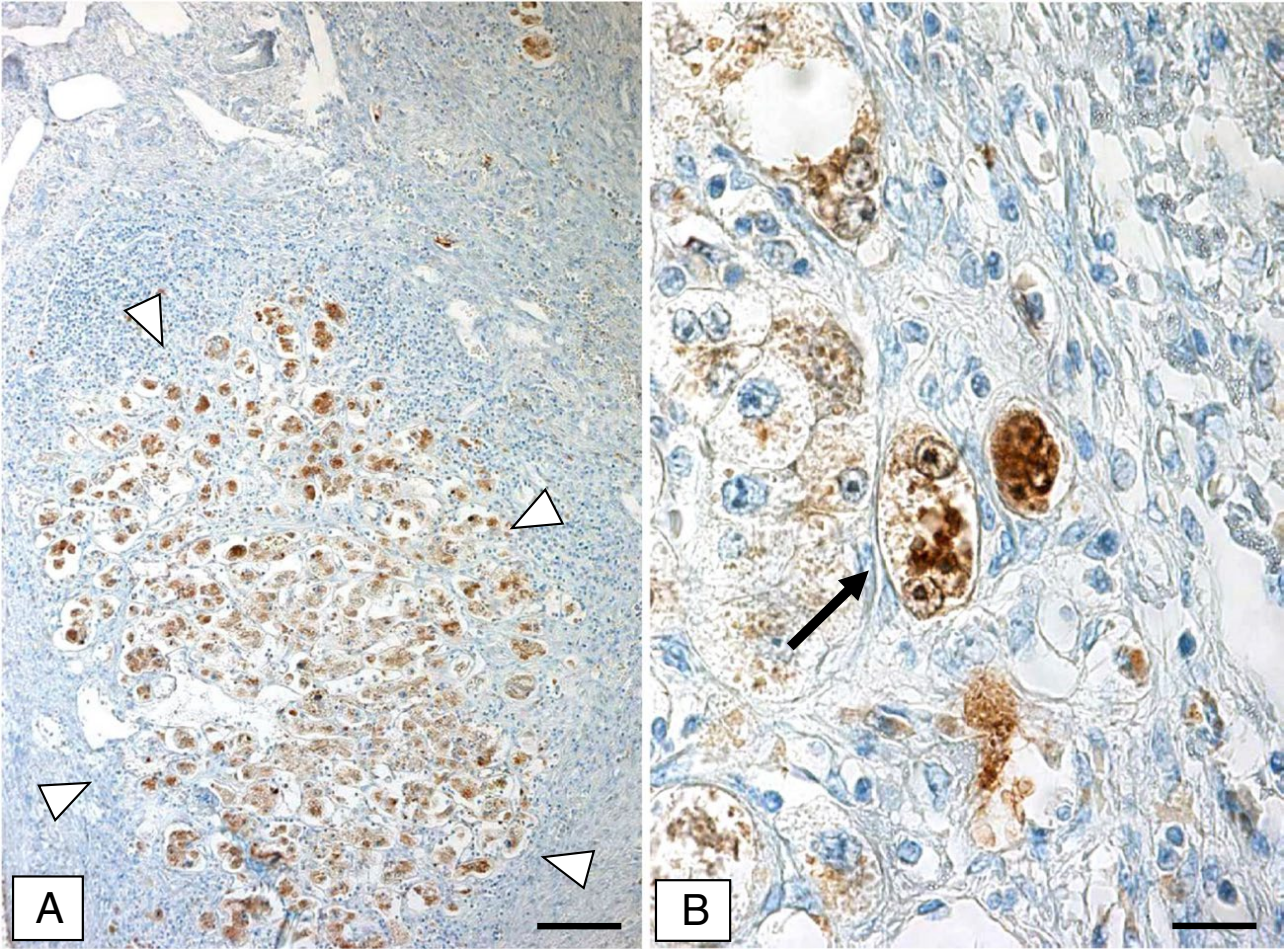
Primary sclerosing cholangitis 4 (cirrhosis). **a** Low magnification of cirrhotic nodule with p16-positive hepatocytes, some of which contain MDBs. **b** p16-positive enlarged hepatocytes containing MDB inclusions (immunohistochemistry using antibodies to p16). Bars: **a** 80 μm; **b** 20 μm

Regarding p16 immunoreactivity, the specimens resembled their alcohol-related counterparts. MDB-containing hepatocytes and MDB inclusions were consistently and strongly p16-positive. Varying numbers of hepatocytes and ductules displayed faint positive cytoplasmic and nuclear p16 immunostaining.

Antibodies to p21 revealed nuclear staining of a variable number of hepatocytes and biliary epithelia. Like in ASH and MASH (described above), some, but not all, MDB-containing hepatocytes displayed p21-positive nuclei, whereas MDBs were consistently p21-negative.

### Wilson’s disease (Table 1)

Liver histology revealed pronounced portal and septal fibrosis or cirrhosis and enlarged hepatocytes with low to moderate macrovesicular steatosis and nuclear vacuolation, as well as varying degrees of septal lymphocytic infiltration. Some ballooned hepatocytes mostly in paraseptal position contained either well-formed dense or less distinct and more granular MDBs. Staining for copper and copper-associated protein revealed positive results.

p16 antibodies revealed faint but specific immunostaining of nuclei and cytoplasm of a variable number of hepatocytes and more pronounced nuclear and cytoplasmic immunoreactivity of ductules. Nuclei and cytoplasm of MDB-containing hepatocytes as well as MDBs were strongly p16-positive.

p21-specific immunohistochemistry revealed nuclear staining of a variable number of hepatocytes and biliary epithelia without relationship to the presence of MDBs. MDBs were negative.

### Hepatocellular carcinoma (Figs. 3 and 4; Table 1)

Four hepatocellular carcinomas (HCCs) histologically resembling the steatohepatitic variant [16] were included in our study. In one of them, tumor cells contained intracellular hyaline bodies (IHBs) in addition to MDBs. In two cases, adherent non-neoplastic cirrhotic liver containing MDBs was present.

**Fig. 3:**
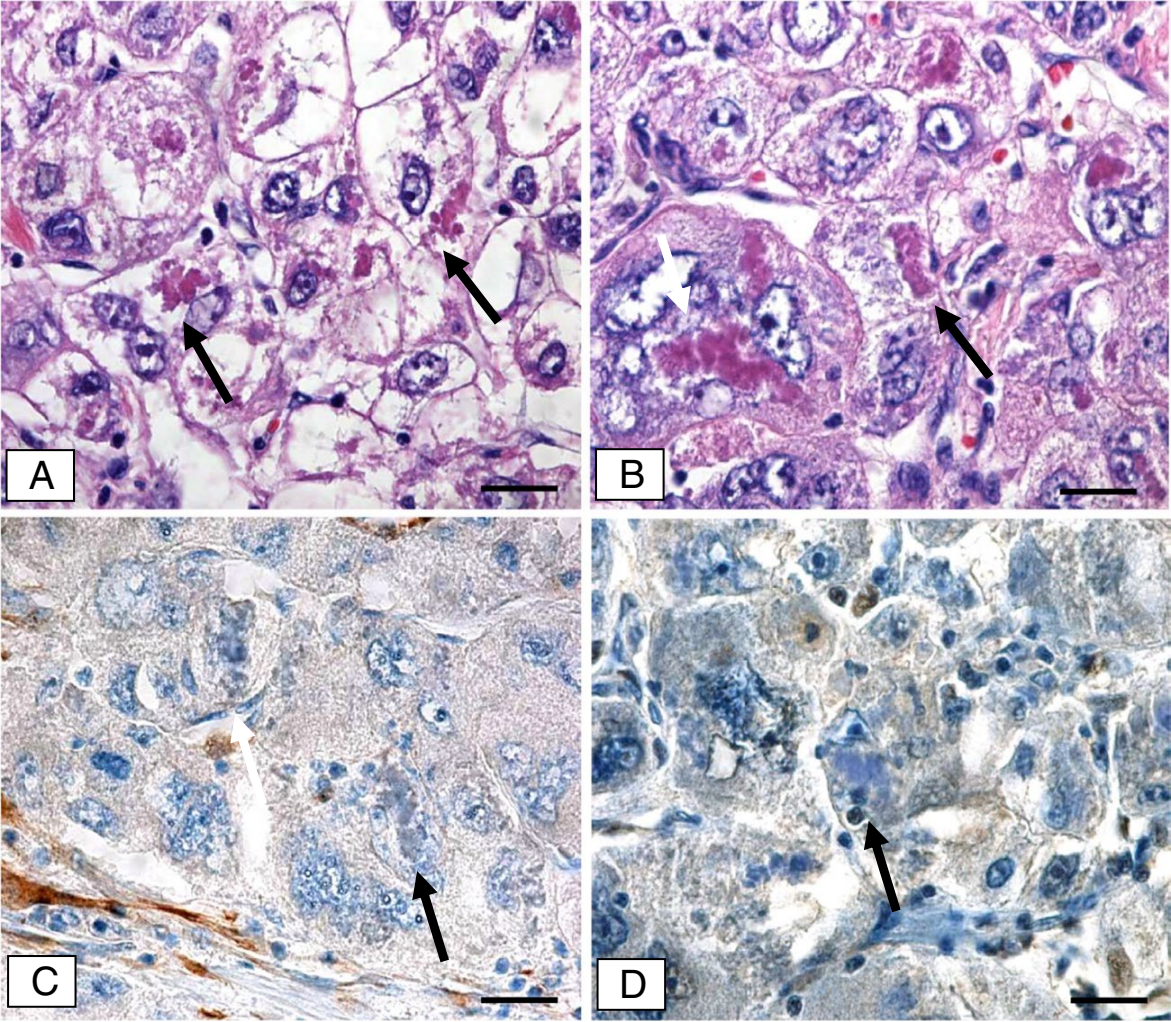
Hepatocellular carcinoma of steatohepatitic type expressing MDBs. **a** Tumor cells with weakly stained cytoplasm, some of which contain dense MDBs (arrows). **b** Polymorphic carcinoma cells containing MDBs (arrows). **c** Tumor cells and MDBs lack p16-specific immunostaining (arrows). Some elongated stroma cells at the lower left corner of the figure are p16-positive (immunohistochemistry using antibodies to p16; arrows). **d** Tumor cells and MDBs lack p21-immunostaining (immunohistochemistry using antibodies to p21). Bars: **a**–**d** 20 μm

**Fig. 4:**
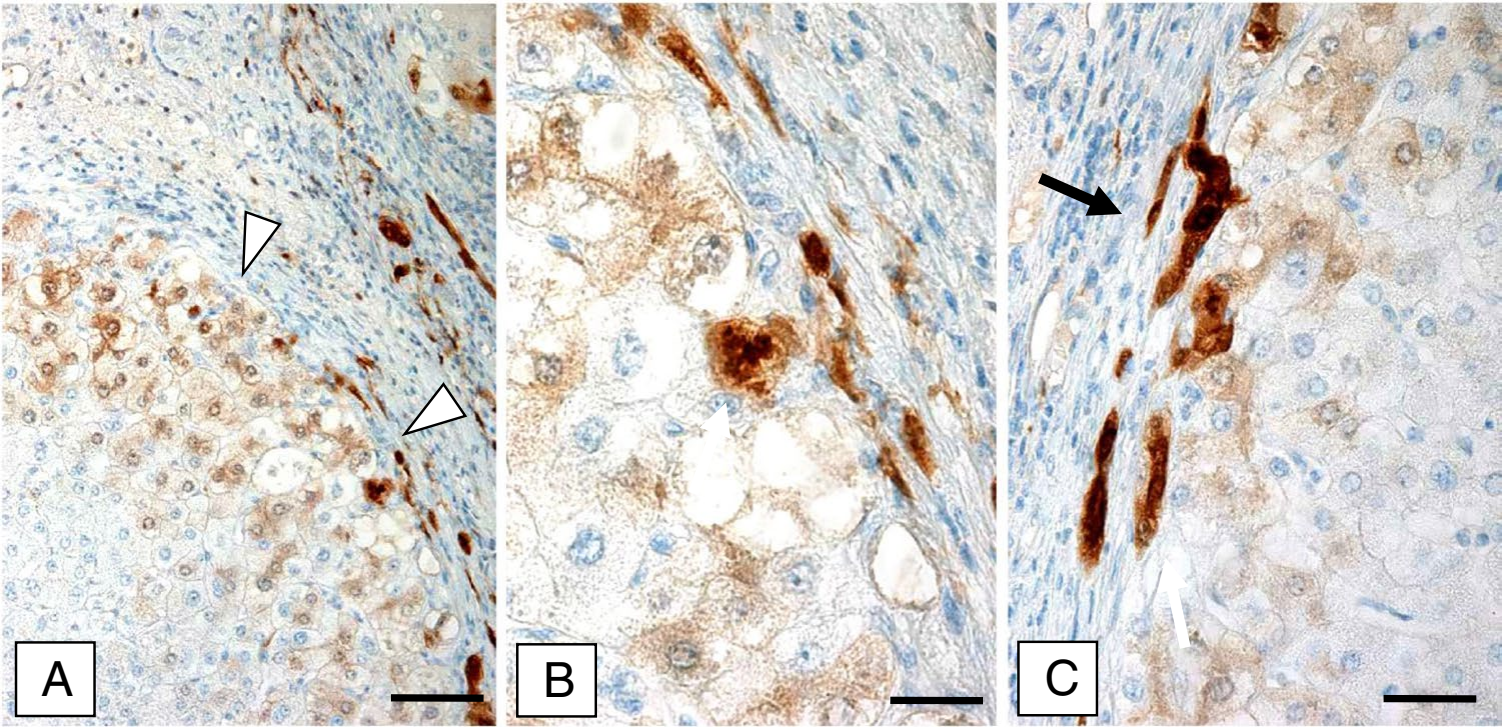
Non-neoplastic liver parenchyma surrounding the hepatocellular carcinoma contains areas of hepatocytes expressing p16 (**a**; arrow-heads). **b** p16-positive hepatocyte containing a positive MDB is shown at higher magnification (arrow). **c** Elongated ductules at the connective tissue/parenchyma border display nuclear and cytoplasmic p16 positivity (immunohistochemistry using antibodies to p16; arrows). Bars: **a** 70 μm; **b** 20 μm; **c** 15 μm

Tumor cells with and without MDBs as well as MDBs were consistently p16-negative. The p16-related immunoreactivity in the surrounding cirrhotic liver resembled that described above. MDB-containing hepatocytes as well as MDBs were strongly p16-positive, whereas IHBs were negative.

Antibodies to p21 revealed positive nuclear immunostaining in a variable number of tumor cells including MDB-containing ones. MDBs were p21-negative in tumor cells as well as in non-neoplastic hepatocytes of the surrounding cirrhotic liver.

## Discussion

The stress-activated cyclin-dependent kinase inhibitors p21 and p16 are regarded (although not absolute specific) as markers of cellular senescence. Their interaction with CDK2 (p21) and CDK4/6 (p16), respectively, maintains Rb protein family members in a hypophosphorylated state responsible for long-lasting proliferation arrest. While p21 usually participates in the initiation of senescence, p16 maintains durable growth arrest, pointing to the existence of differently regulated phases of senescence [10–13]. Our study revealed the expression of p16 and p21 in hepatocytes and ductular epithelia in steatohepatitis (ASH and MASH), chronic cholangiopathies, and Wilson’s disease, which are often associated with MDB formation [1–3]. It is shown that cytoplasm and nuclei of non-neoplastic MDB-containing hepatocytes as well as MDB inclusions were strongly positive for p16, which confirms our previous results obtained in focal nodular hyperplasia [17]. Thus, our findings suggest that MDB formation is related to cellular senescence. This agrees with the fact that cellular senescence is a feature of a variety of chronic liver diseases [10, 11, 18–23]; see there for further information]. However, the correlation of nuclear p21 immunostaining with MDB expression was less obvious since we observed p21-positive as well as p21-negative nuclei in MDB-containing hepatocytes. This may be related to the different cellular pathways involved in p16 and p21 activation and regulation.

The chemical composition of MDBs provides hints to their pathogenesis (this may also be more generally relevant to cytoplasmic inclusion bodies associated with a variety of chronic degenerative neurological disorders; see also 6). Stress-related misfolded proteins as major MDB components may result from their elevated occurrence in response to stress followed by impaired degradation and aggregation2. This can explain the fact that MDB formation is not a uniform phenomenon in the liver parenchyma: the appearance of visible MDBs in hepatocytes most likely depends on the specific metabolic constellation of the individual cells, i.e., the relationship between protein synthesis and degradation in agreement with their well-known heterogeneity within the liver [24]. Interestingly, MDBs present in tumor cells of the steatohepatitic HCC variant [16] in contrast to that present in surrounding non-neoplastic liver were p16-negative. The reason for this discrepancy to non-neoplastic liver is unclear; it is, however, possible that in neoplastic hepatocytes due to metabolic differences, stress pathways leading to MDB formation are independent of p16. Inactivation of the p16 pathway with loss of p16 expression, e.g., due to hypermethylation of the promoter region, is a frequent event in HCCs [25–29]. This observation and the role of p21 in this context require further studies.

Chronic stress is a major inducer of cellular senescence [10–13]. Senescent cells are not only long-lived and resistant to apoptosis but also metabolically modified resulting in the senescence-associated secretory phenotype (SASP) with production of cytokines, chemokines, and vasoactive substances, which are responsible for inflammation and fibrosis [10–13]. In this respect, however, p16 and p21 differ as shown by Sturmlechner et al. [30]. Both activate distinct target genes responsible for secretomes of different biological properties. p21 also activates genes implicated in immunosurveillance by recruiting macrophages and cytotoxic T lymphocytes to stressed cells, whereas p16-associated SASP has less immune-related effects. Although cellular senescence primarily resembles a protective approach, particularly with respect to prevention of neoplastic transformation, a fibrosing inflammatory process related to p16 expression may be initiated and maintained [12, 13]. The p21 pathway may also contribute to the morphologic phenotype of the disease.

The association of MDB development with senescence and adaptation to chronic stress is supported by the following observations: (i) aging enhances susceptibility to MDB formation in humans and mice [5]; (ii) MDBs develop in certain long-lasting (chronic) liver diseases in humans and experimental animals, which are often associated with oxidative stress and cellular senescence [11, 12]; (iii) MDB-containing hepatocytes are viable, ballooned with nuclear/nucleolar enlargement typical of senescent cells, and prefer areas in the liver with known increased oxidative stress [2, 31]; (iv) calcium and related cellular processes are involved in the regulation of cellular senescence [32], and increased intracellular calcium concentrations could be responsible for the calcium-dependent stabilization of MDB components (particularly Ks) by transglutaminase-induced cross-linking [2, 3]. (v) MDB-containing hepatocytes may be associated with inflammation consisting of neutrophils and mononuclear cells (“satellitosis”), pericellular fibrosis, and production of TNFα, which could resemble SASP activity [2, 33].

Regarding senescence of biliary epithelium, our results confirm reports by other authors. Senescence, particularly of “atypical” ductules arranged in anastomosing cords with poorly defined lumina [34, 35], may also contribute to liver fibrosis in agreement with the fact that in chronic liver diseases ductular reaction correlates with disease progression [19, 20, 22, 23, 36–41].

**Fig. 5:**
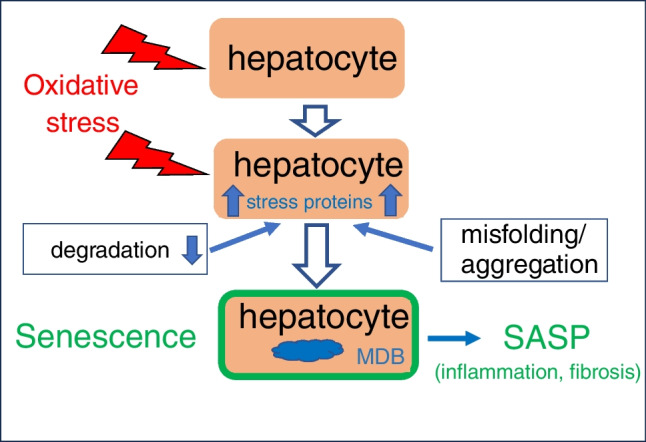
Schematic summary of proposed MDB pathogenesis (MDB, Mallory-Denk body; SASP, senescence-associated secretory phenotype)

In diagnostic histopathology, detection of abnormalities of cells and tissues and their grading provides clinically relevant information regarding etiology, pathogenesis, prognosis, and therapy. In acute liver injury, pathologic features, e.g., degenerative changes, steatosis, apoptosis, necrosis, inflammation, and fibrosis, result from exogenous or endogenous (e.g., genetic, chemical, physical, and microbial) insults, and their disappearance upon therapy means therapeutic success. However, abnormal morphologic features, as shown here with MDBs as example, may also resemble an adaptive response to chronic stress leading to cellular senescence with its consequences, i.e., inflammation- and fibrosis-prone secretome (Fig. 5). Thus, senescence can be regarded as “double-edged sword”: on the one hand, it may be the result of cellular defense, even providing protection against neoplastic transformation related to DNA damage, and, on the other, it causes further damage by inflammation and fibrosis. Moreover, MDBs as poorly soluble intracellular protein aggregates can interfere with cellular transport processes, proteasomal function, or may deprive the cell of vital components [3]. As a consequence, formation of MDBs can be seen not only as “morphological biomarker” associated with cellular stress and aging but also as product of accumulation of misfolded proteins, which in turn may enhance aging-related cellular responses as indicated by the p16 positivity. This establishes a new diagnostic potential of MDBs, particularly because they are independently associated with fibrosis progression and finally liver failure [42–44].

## Abbreviations

p21: CDK inhibitor 1/p21^Cip1^
p16: CDK inhibitor 2/p16^Ink4a^
H&E: Hematoxylin and eosin
K: Keratin
ASH: Alcohol-related steatohepatitis
MASH: Metabolic dysfunction-associated steatohepatitis
SASP: Senescence-associated secretory phenotype
MDB: Mallory-Denk body
HCC: Hepatocellular carcinoma
